# The Pharmacological Potential of Non-ribosomal Peptides from Marine Sponge and Tunicates

**DOI:** 10.3389/fphar.2016.00333

**Published:** 2016-10-25

**Authors:** Shivankar Agrawal, Alok Adholeya, Sunil K. Deshmukh

**Affiliations:** TERI–Deakin Nano Biotechnology Centre, The Energy and Resources InstituteNew Delhi, India

**Keywords:** marine ecosystem, sponge, tunicates, marine natural products, non-ribosomal peptides, pharmacology

## Abstract

Marine biodiversity is recognized by a wide and unique array of fascinating structures. The complex associations of marine microorganisms, especially with sponges, bryozoans, and tunicates, make it extremely difficult to define the biosynthetic source of marine natural products or to deduce their ecological significance. Marine sponges and tunicates are important source of novel compounds for drug discovery and development. Majority of these compounds are nitrogen containing and belong to non-ribosomal peptide (NRPs) or mixed polyketide–NRP natural products. Several of these peptides are currently under trial for developing new drugs against various disease areas, including inflammatory, cancer, neurodegenerative disorders, and infectious disease. This review features pharmacologically active NRPs from marine sponge and tunicates based on their biological activities.

## Introduction

Nature provides a wide and structurally diverse array of active biomolecules that have proved vital for the development of novel pharmaceuticals. The marine world, covering more than 70% of the Earth's surface, is the home of tremendous biodiversity. Due to very diverse oceanic environments, marine organisms have developed the capacity to produce unique compounds (Steele, 1985; Mehbub et al., 2014). This rich and unprecedented chemo diversity of marine natural products provides an unlimited resource of novel biomolecules in the field of drug development. The importance of marine metabolites in current drug research is driven by the fact, that during 1981–2002, around half of US FDA-approved drugs consisted of either marine metabolites or their synthetic analogs (Vinothkumar and Parameswaran, 2013). Interestingly, the majority of these natural products involved in clinical or preclinical trials are produced by invertebrates, that is, sponges, tunicates, bryozoans, or molluscs. Sixty per cent of these natural products belong to non-ribosomal peptide (NRP) families, which are biosynthesized by poly-functional mega-synthases called NRP synthetases (NRPSs) (Finking and Marahiel, 2004; Mehbub et al., 2014). The excellent binding properties, low off-target toxicity, and high stability of NRPs make them a promising molecule for development of new therapeutics. Currently, only a handful of NRPs are used as drug (Table 1).

**Table 1:** **NRPs-based drugs in market**.

**Compound**	**Biosynthetic class of agent**	**Source**	**Disease/molecular target**	**Reference**
Polymyxin B	Polypeptides	*Bacillus polymyxa*	Antibiotic/Alters bacterial outer membrane	Paulus and Gray, 1964
Pristinamycin	Depsipeptide	*Streptomyces. pristinaespiralis*	Antibiotic/protein synthesis inhibitor	de Crécy-Lagard et al., 1997
Gramicidin	Linear pentadecapeptide	*Bacillus bovis*	Antibiotic/Alters bacterial outer membrane	Kleinkauf and von Döhren, 1995
Bacitracin	Cyclic peptide	*Bacillus subtilis*	Antibiotic/dephosphorylation of C55-isoprenyl pyrophosphate	Johnson et al., 1945
Capreomycin	Cyclic peptide	*Streptomyces capreolus*	Antibiotic/protein synthesis inhibitor	Stark et al., 1962
Teicoplanin	Glycopeptide	*Actinoplanes teichomyceticus*	Antibiotic/inhibit cell wall synthesis	Somma et al., 1984
Vancomycin	Glycopeptide	*Amycolatopsis orientalis*	Antibiotic/inhibit cell wall synthesis	Van Wageningen et al., 1998
Cephalosporin C	β-lactam	*Acremonium* sp.	Antibiotic/Alters bacterial outer membrane	Abraham and Newton, 1961
Oritavancin	–	Semi synthetic	Antibiotic/disrupts the cell membrane	Domenech et al., 2009
Bleomycin	Hybrid peptide	*Streptomyces verticillus*	Antibiotic/inhibition of DNA synthesis	Umezawa et al., 1966
Daptomycin	Lipopeptide	*Streptomyces roseosporus*	Antibiotic/disrupts the cell membrane	Miao et al., 2005
Cyclosporine A	Cyclic peptide	*Tolypocladium inflatum*	Immunosuppressant /lower the activity of T cells	Murthy et al., 1999
Actinomycin D	Polypeptide	*Streptomyces* sp.	Antitumor/inhibit transcription	Waksman and Woodruff, 1940
Romidepsin	Depsipeptide	*Chromobacterium violaceum*	Antitumor/Histone deacetylase inhibitor	Ueda et al., 1994

Marine sponges (*Phylum porifera*) represent the most primitive multicellular animals, with origins dating back to the Precambrian era (Hentschel et al., 2002). There are about 9000 reported species of sponges and (perhaps twice as many unreported species) available in the ocean (Brusca et al., 1990; Wörheide et al., 2005). These have been broadly categorized in 3 classes : Calcarea (5 orders and 24 families), Demospongiae (15 orders and 92 families), and Hexactinellida (6 orders and 20 families). Till date, more than 5300 different natural products have been isolated from marine sponges, and each year more than 200 additional new metabolites are being discovered (Laport et al., 2009; Mehbub et al., 2014). There are several sponge derived metabolites currently available in market and many in clinical studies (Table 2).

**Table 2:** **Sponge secondary metabolites that are FDA-approved agents in clinical trial (Mayer et al., 2010; Newman and Cragg, 2016)**.

**Compound**	**Biosynthetic class of agent**	**Source**	**Disease/molecular target**	**Clinical status**
Cytarabine (Ara-C)	Nucleoside	*Cryptotethya crypta*	Cancer/DNA polymerase	FDA approved
Vidarabine (Ara-A)	Nucleoside	*C. crypta*	Antiviral viral/DNA polymerase I	FDA approved
Eribulin mesylate (E7389)	Complex polyketide	*Lissodendoryx* sp.	Cancer/microtubules	FDA approved
Hemiasterlin derivative (E7974)	Modified linear tripeptide (NRPS-PKS)	*Cymbastella* sp.	Cancer/microtubules	Phase I
Discodermolide	Polyketide	*Discodermia dissolute*	Cancer/microtubules	Phase I
Bengamide derivative (LAF389)	Mixed PKS/NRP	*Jaspis* sp.	Cancer/methionine aminopeptidases	Phase I
Spongistatin 1	Macrocyclic lactone polyether	*Hyrtios erecta*	Cancer/microtubules	Preclinical
Manoalide	Sesterterpene	*Luffariella variabilis*	Inflammation/inhibition of Phospholipase A2	Preclinical
Salicylhalimides A	Polyketide	*Haliclona* sp.	Cancer/microtubules	Preclinical
Laulimalide	Polyketide	*Cacospongia mycofijiensis*	Cancer/microtubules	Preclinical
Peloruside A	Polyketide	*Mycale hentscheli*	Cancer/microtubules	Preclinical

It is proposed that some of the bioactive compounds isolated from sponges are produced by functional enzyme clusters originated from the sponges and their associated microorganisms (Laport et al., 2009; Thomas et al., 2010). It has been observed that bacterial phyla such as *Proteobacteria, Nitrospira, Cyanobacteria, Bacteriodetes, Actinobacteria, Chloroflexi, Planctomycetes, Acidobacteria, Poribacteria*, and *Verrucomicrobia* besides members of the domain *Archaea* are most sponge-associated bacterial community (Hentschel et al., 2002; Olson and McCarthy, 2005). However, fungi and microalgae also symbiotically inhabit sponges. It has been recognized that one host sponge can possess diverse symbionts. For example, unicellular heterotrophic bacteria, unicellular cyanobacteria, and filamentous heterotrophic bacteria all grow together in sponge *Theonella swinhoei* (Bewley et al., 1996). Likewise, a sponge belonging to *Aplysina* includes heterogeneous bacteria *Bacillus* sp., *Micrococcus* sp., *Arthrobacter* sp., *Vibrio* sp., *Pseudoalteromonas* sp., and so on (Hentschel et al., 2001). Sponge *Rhopaloeides odorabile has* β*-Proteobacteria*, γ*-Proteobacteria, Cytophaga, Actinobacteria, and* green sulfur bacteria (Webster et al., 2001). Besides this, species-specific symbiotic relationship has also been observed. For example, sponge *T. swinhoei* and δ-*proteobacteria* have shown a specific association with each other (Schmidt et al., 2000). A species of α-*proteobacteria* dominates in sponge *R. odorabile* over various habitats but is not detected from seawater, which strongly suggests that the symbiont is species specific (Lee Y. K. et al., 2001). On the other hand, one symbiont occurs commonly in various sponges from different regions indicating its wide host range (Wilkinson et al., 1981). For example, cyanobacteria *Aphanocapsa* sp., *Phormidium* sp., or *Oscillatoria spongeliae* are found in numerous sponges (Wilkinson, 1978). Symbiotic associations between sponges and marine microorganisms might be involved in nutrient acquisition, stabilization of sponge skeleton, processing of metabolic waste, and secondary metabolite synthesis. It is assumed that symbiotic marine microorganisms harbored by sponges are the original producers of some of these bioactive compounds (Newman and Hill, 2006). For example, antibiotic polybrominated biphenyl ether isolated from the sponge *Dysidea herbacea* (Demospongiae) are actually produced by endosymbiotic cyanobacterium *O. spongeliae* (Unson et al., 1994). A symbiotic bacterium *Micrococcus* sp. produces diketopiperazines previously ascribed to the host sponge *Tedania ignis* (Stierle et al., 1988). Another symbiotic bacterium *Vibrio* sp. produces brominated biphenyl ethers formerly attributed to the host sponge *Dysidea* sp. (Elyakov et al., 1991). Symbiotic bacterium *Vibrio* sp. produces an anti-Bacillus peptide andrimid that was found in the sponge *Hyatella* sp. extract (Oclarit et al., 1994). Antimicrobial activity is detected in *Micrococcus luteus* isolated from the sponge *Xestospongia* sp. (Bultel-Poncé et al., 1998). Antimicrobial compounds such as quinolones and phosphatidyl glyceride are isolated from a *Pseudomonas* sp. collected at the surface of the sponge *Homophymia* sp. (Bultel-Poncé et al., 1999). However, the mutual mechanism between sponge and its microbial associate, in metabolite production, is not well-understood. Thus, it is extremely relevant to highlight the therapeutic potential of various secondary metabolites synthesized by the microbial flora inhabiting sponges. This is because they open up the possibility of providing a continuous supply of the biologically active compounds by laboratory cultivation of the producer (Thomas et al., 2010).

Tunicates include a wide variety of invertebrates that are classified within the *Phylum chordata* based on presence of a larval notochord during early development. Tunicates contains about 2150 described species that are divided into 4 classes: *Ascidiacea* (*Aplousoobranchia, Phlebobranchia, Stolidobranchia*) *Thaliacea* (*Pyrosomida, Doliolida, Salpida*), *Appendicularia* (*Larvacea*), and *Sorberacea* (Ruppert and Fox, 2004). Amongst these, *Ascidacea* (commonly known as the ascidians) are highly studied due to their biologically active metabolites that serve as antineoplastic agents. Geranyl hydroquinone was first ascidian metabolite isolated from *Aplidium* sp. which displayed chemo protective activity against some forms of leukemia, rous sarcoma, and mammary carcinoma in test animals (Fenical, 1976) (Menna, 2009). Since then, ascidians are known as the source of numerous marine natural products. The biologically active metabolites originated from tunicates which are approved by FDA or in clinical trials along with their biological properties are given in Table 3.

**Table 3:** **Tunicate secondary metabolites that are FDA-approved agents or in clinical trial (Mayer et al., 2010; Newman and Cragg, 2016)**.

**Compound**	**Biosynthetic class of agent**	**Source**	**Disease/molecular target area**	**Clinical status**
Trabectedin (ET-743) (EU registered only)	NRPS-derived alkaloid	*Ecteinascidia turbinata*	Cancer/minor groove of DNA	FDA approved
Plitidepsin (Aplidine)	Cyclic depsipeptide	*Aplidium albicans*	Cancer/Rac1 and JNK activation	Phase III
Trabectedin analog (PM01183)	NRPS alkaloid	*E. turbinata*	Cancer/minor groove of DNA, nucleotide excision repair	Phase I
Vitilevuamide	NRPS	*Didemnum cuculiferum* and *Polysyncranton lithostrotum*	Cancer/microtubules	Preclinical

To date, significant biological activities, such as antimicrobial, anticancer, neurotoxic, antiprotozoal and their associated cellular targets have been reported for several NRPs from the marine sponges and tunicates. These NRPs have unique structures as compared with those from other sources. It is this attribute that makes marine sponge- and tunicate-derived NRPs highly attractive as potential drug and molecular probes. In this review, we survey the discoveries of NRPs derived from marine sponges and tunicates, which have shown *in vivo* efficacy or potent *in vitro* activity against various human diseases. Our objective is to highlight NRPs that have the greatest potential to be clinically useful. The details of sponge- and tunicate-derived NRPs along with biological properties is given Table 4.

**Table 4:** **Biological activities of NRPs isolated from marine sponges and tunicates**.

**NRPs**	**Chemical class**	**Origin**	**Disease/target**	**Biological active value (IC_50_/GI_50_/ID_50_/ED_50_)**	**Reference(s)**
Miraziridine A **(1)**	Linear penta peptide	*Theonella* aff. *mirabilis*	Cancer/inhibit protease cathepsin B	1.4 μg/mL	Nakao et al., 2000
Haligramides A-B **(2–3)**	Cyclic hexapeptides	*Haliclona nigra*	Cancer/A-549 (lung) HCT-15(colon) SF-539 (CNS) SNB-19 (CNS)	5.17–15.6 μg/mL 3.89–8.82 μg/mL	Rashid et al., 2000
Prepatellamide A **(4)**	Cyclic peptide	*Lissoclinum patella*	Cancer/P388 murine leukemia cell lines	5 μg/mL	Fu et al., 2000
Tamandarins A-B **(5–6)**	Depsipeptides	*Didemnid ascidian*	Cancer/pancreatic carcinoma BX- PC3, prostatic cancer DU-145, head and neck carcinoma UMSCC10b	1.79, 2.00 μg/mL 1.36, 1.53 μg/mL 0.99, 1.76 μg/mL	Vervoort et al., 2000
Microsclerodermins F–I **(7–10)**	Cyclic peptides	*Microscleroderma* sp.	Cancer/HCT-116 cell line	1.8, 2.4, 1.0, and 1.1 μg/mL	Qureshi et al., 2000
Wainunuamide **(11)**	Cyclic hexapeptide	*Stylotella aurantium*	Cancer/A2780 ovarian, K562 leukemia cancer cells	19.15 and 18.36 μg/mL	Tabudravu et al., 2001
Leucamide A **(12)**	Cyclic hexapeptide	*Leucetta microraphis*	Cancer/Tumor cell lines HM02, HepG2, Huh7	5.2 μg/mL 5.9 μg/mL 5.1 μg/mL	Kehraus et al., 2002
Axinellin C **(13)**	Cyclic octapeptide	*S. aurantium*	Cancer/A2780 ovarian, K562 leukemia cancer cells	13.17 and 4.46 mg/mL	Tabudravu et al., 2002
Milnamide D **(14)**	Linear peptide	*Cymbastela* sp.	Cancer/HCT-116 cells	66 nM	Chevallier et al., 2003
Kapakahines E–G **(15–17)**	–	*Cribrochalina olemda*	Cancer/P388 murine leukemia cells	5.0 μg/mL	Nakao et al., 2003
Didmolamides A- B **(18–19)**	Cyclic hexapeptides	*Didemnum molle*	Cancer Tumor cell lines (A549, HT29, and MEL28)	10–20 μg/mL	Rudi et al., 2003
Bistratamides E–J **(20- 25)**	Cyclic hexapeptides	*Lissoclinum bistratum*	Cancer/Human colon tumor (HCT-116) cell line	3, 7.9; 4, 28; 5, 5; 6, 1.7; 7, 9; 8, 1 μg/mL	Perez and Faulkner, 2003
Milnamide C **(26)**	–	*Auletta* sp.	Cancer/MDA-MB-435 cancer cells	3.2 × 10^−1^ μg/mL	Sonnenschein et al., 2004
Scleritodermin A **(27)**	Cyclic peptide	*Scleritoderma nodosum*	Cancer	<2 μM	Schmidt et al., 2004
Microcionamides A-B **(28–29)**	–	*Clathria abietina*	Cancer/Human breast tumor cell lines MCF-7 and SKBR-3	125 and 98 nM 177 and 172 nM	Davis et al., 2004
Kendarimide A **(30)**	Linear peptide	*Haliclona* sp.	Cancer/KB-C2 cells	–	Aoki et al., 2004
Phakellistatin 14 **(31)**	Cycloheptapeptide	*Phakellia* sp.	Cancer/Murine lymphocytic leukemia P388 cell line	5 μg/mL	Pettit and Tan, 2005
Polytheonamides A-B **(32–33)**	Polypeptides	*T. swinhoei*	Cancer/P388 murine leukemia cells	78, 68 pg/mL	Hamada et al., 2005
Neopetrosiamides A- B **(34–35)**	Tricyclic peptides	*Neopetrosia* sp.	Cancer	6 μg/mL	Williams et al., 2005
Seragamides A–F **(36–37)**	Depsipeptides	*Suberites japonicus*	Cancer	0.01, 0.02, 0.01, 0.01, and 0.04 mg/mL	Tanaka et al., 2006
Theopapuamide **(38)**	Cyclic depsipeptide	*T. swinhoei*	Cancer/CEM-TART HCT-116 cell lines	0.5 μM 0.9 μM	Ratnayake et al., 2006
Azumamide A- E **(39–47)**	Cyclotetrapeptides	*Mycale izuensis*	Cancer	–	Maulucci et al., 2007
Callyaerin G **(48)**	Cyclic peptide	*Callyspongia aerizusa*	Cancer/Mouse lymphoma cell line (L5178Y) and HeLa cells	0.53 and 5.4 ug/mL	Ibrahim et al., 2008
Stylopeptide 2 **(49)**	Cyclodecapeptide	*Stylotella* sp.	Cancer/BT-549 and HS 578T breast cancer cell lines	–	Brennan et al., 2008
Ciliatamides A-C **(50–52)**	Lipopeptides	*Aaptos ciliata*	Cancer/HeLa cells	50, 4.5, and 50 μg/mL	Nakao et al., 2008
Diazonamides C–E **(53–55)**	Macrocyclic peptides	*Diazona* sp.	Cancer/Human tumor cell lines (A549, HT29, MDA-MB 231)	2.2, 2.9, 8.0 μg/mL 1.8, 2.9, 5.2 μg/mL 2.2, 3.1, 9.0 μg/mL	Fernández et al., 2008
Rolloamide A- B **(56–57)**	Cyclic heptapeptides	*Eurypon laughlini*	Cancer	0.4−5.8 μM	Williams et al., 2009
Euryjanicin A **(58)**	Cycloheptapeptide	*Prosuberites laughlini*	Cancer	–	Vicente et al., 2009
Callyaerin A–F **(59–64)** and H **(65)**	Cyclic peptides	*C. aerizusa*	Cancer/L5178Y cell line	0.39 and 0.48 μM	Ibrahim et al., 2010
Papuamides E-F **(66–67)**	Depsipeptides	*Melophlus* sp.	Cancer/Brine shrimp	92 and 106 μg/mL	Prasad et al., 2011
Stylissamide X **(68)**	Octapeptide	*Stylissa* sp.	Cancer/HeLa cells	0.1 μM to 10 μM	Arai et al., 2012
Gombamide A **(69)**	Hexapeptide	*Clathria gombawuiensis*	Cancer/K562 and A549 cell lines	6.9 and 7.1 μM	Woo et al., 2013
Microspinosamide **(70)**	Cyclic depsipeptide	*Sidonops microspinosa*	HIV	0.2 μg/mL	Rashid et al., 2001
Neamphamide A **(71)**	Cyclic depsipeptide	*Neamphius huxleyi*	HIV	8 nM	Oku et al., 2004
Mirabamides A-D **(72–75)**	Cyclic depsipeptide	*Siliquariaspongia mirabilis*	HIV	40 and 140 nM, 140 nM and 1.3 μM 190 nM and 3.9 μM	Plaza et al., 2007
Homophymine A **(76)**	Cyclodepsipeptide	*Homophymia* sp.	HIV/PBMC cell line	75 nM	Zampella et al., 2008
Celebeside A-C **(77–79)**	Depsipeptides	*S. mirabilis*	HIV/Colon carcinoma (HCT-116) cells	2.1 and 4.0 μg/mL 1.9 ± 0.4 μg/mL	Plaza et al., 2008
Theopapuamides B–D **(80–82)**					
Mutremdamide A **(83)** Koshikamides C-H (84–89)	Cyclic depsipeptide	*Theonella* sp.	HIV	2.3 and 5.5 μM	Plaza et al., 2010
Ceratospongamide **(90)**	Cyclic heptapeptide	*Sigmadocia symbiotica*	Inflammation	32 nM	Tan et al., 2000
Halipeptin A-B **(91–92)**	Cyclic depsipeptide	*Haliclona* sp.	Inflammation	300 μg/kg (i.p.)	Randazzo et al., 2001
Perthamide C-D **(93–94)**	Cyclopeptide	*T. swinhoei*	Inflammation	–	Festa et al., 2009
Solomonamide A- B **(95–96)**	Cyclic peptide	*T. swinhoei*	Inflammation	–	Festa et al., 2011
Stylissatin A **(97)**	Cyclic peptide	*Stylissa massa*	Murine macrophage RAW264.7	87 μM	Kita et al., 2013
Dicynthaurin **(98)**	–	*Halocynthia aurantium*	Antimicrobial	–	Lee I. H. et al., 2001
Nagahamide A **(99)**	Depsipeptide	*T. swinhoei*	Antibacterial	–	Okada et al., 2002
Plicatamide **(100)**	Octapeptide	*Styela plicata*	Antimicrobial	–	Tincu et al., 2003
Callipeltins F-I **(101–104)**	–	*Latrunculia* sp.	Antifungal/*Candida albicans*	10−4 M	Sepe et al., 2006
Callipeltins J-M **(105–108)**	–	*Latrunculia* sp.	Antifungal/*C. albicans*	4−10 M	D'Auria et al., 2007
Citronamides A- B **(109–110)**	–	*Citronia astra*	Antifungal/*Saccharomyces cerevisiae*	8 μg/mL	Carroll et al., 2009
Renieramide **(111)**	Cyclic tripeptide	*Reniera* sp.	–	–	Ciasullo et al., 2002
Phoriospongins A- B **(112–113)**	Depsipeptide	*Phoriospongia* sp. and *Callyspongia bilamellata*	Nematocidal/*Haemonchus contortus*	100, 194 μg/mL	Capon et al., 2002

## Anticancer NRPs from marine sponges and tunicates (figures 1–6)

Sponge *Theonella* aff. *mirabilis* was the source of linear penta peptide Miraziridine A **(1)**, which showed inhibitory activity on cathepsin B with an IC_50_ value of 1.4 μg/mL (Nakao et al., 2000). Two cyclic hexapeptides, Haligramides A **(2)** and B **(3)**, were isolated from an aqueous extract of the sponge *H. nigra*. Both compounds exhibited cytotoxicity against various cell lines. Haligramide A exhibited cytotoxicity against A-549, HCT-15, SF-539, SNB-19 cell line with IC_50_ values of 5.17, 15.62, 9.00, and 9.08 μg/mL, respectively. Haligramide B, was found to be more active than Haligramide A against A-549, HCT-15, SF-539, SNB-19 cell line with IC_50_ values of 3.89, 8.82, 5.01, and 6.56 μg/mL, respectively (Rashid et al., 2000).

**Figure 1:**
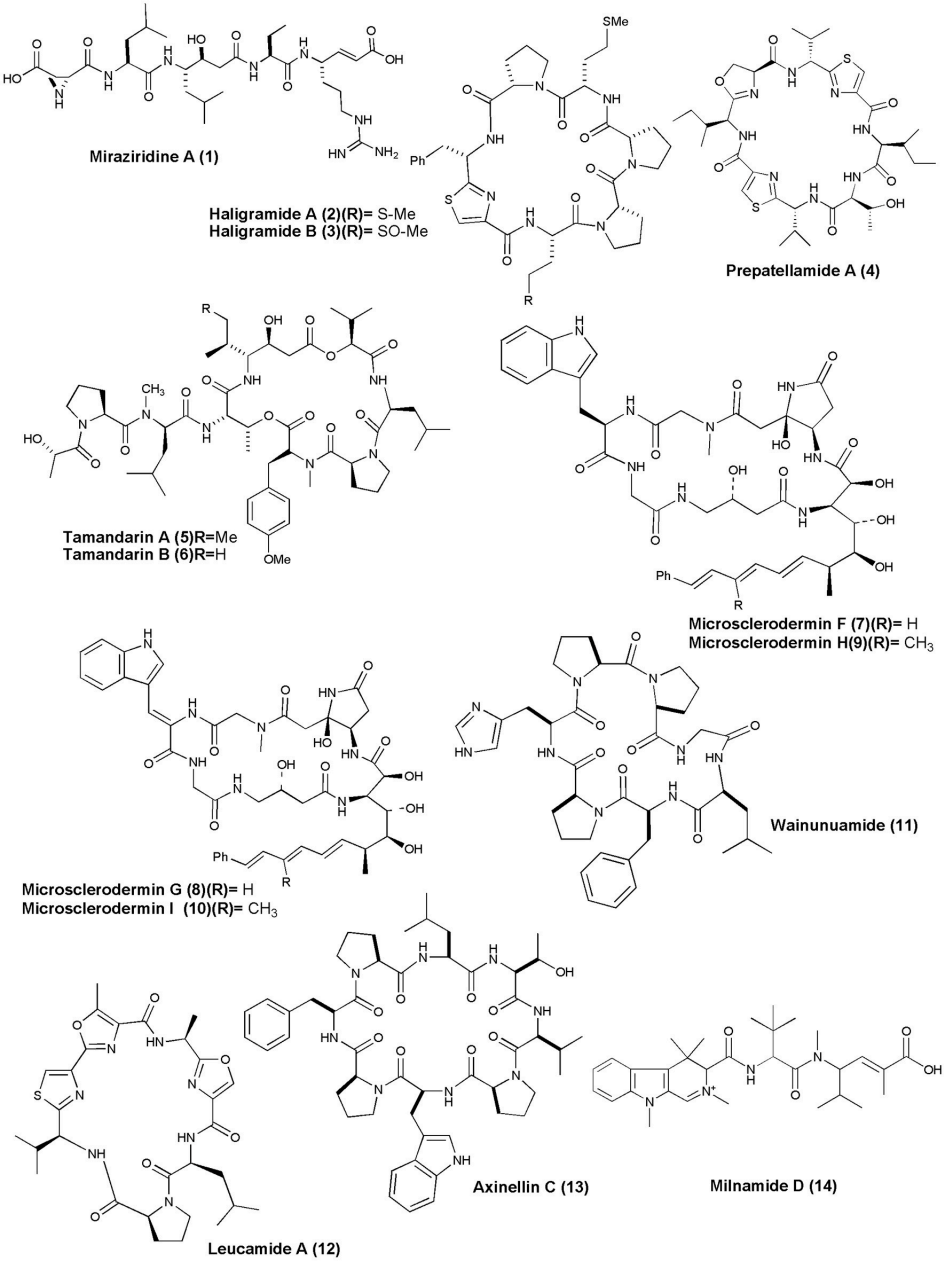
**Structures of anticancer non-ribosomal peptides (1–14)**.

**Figure 2:**
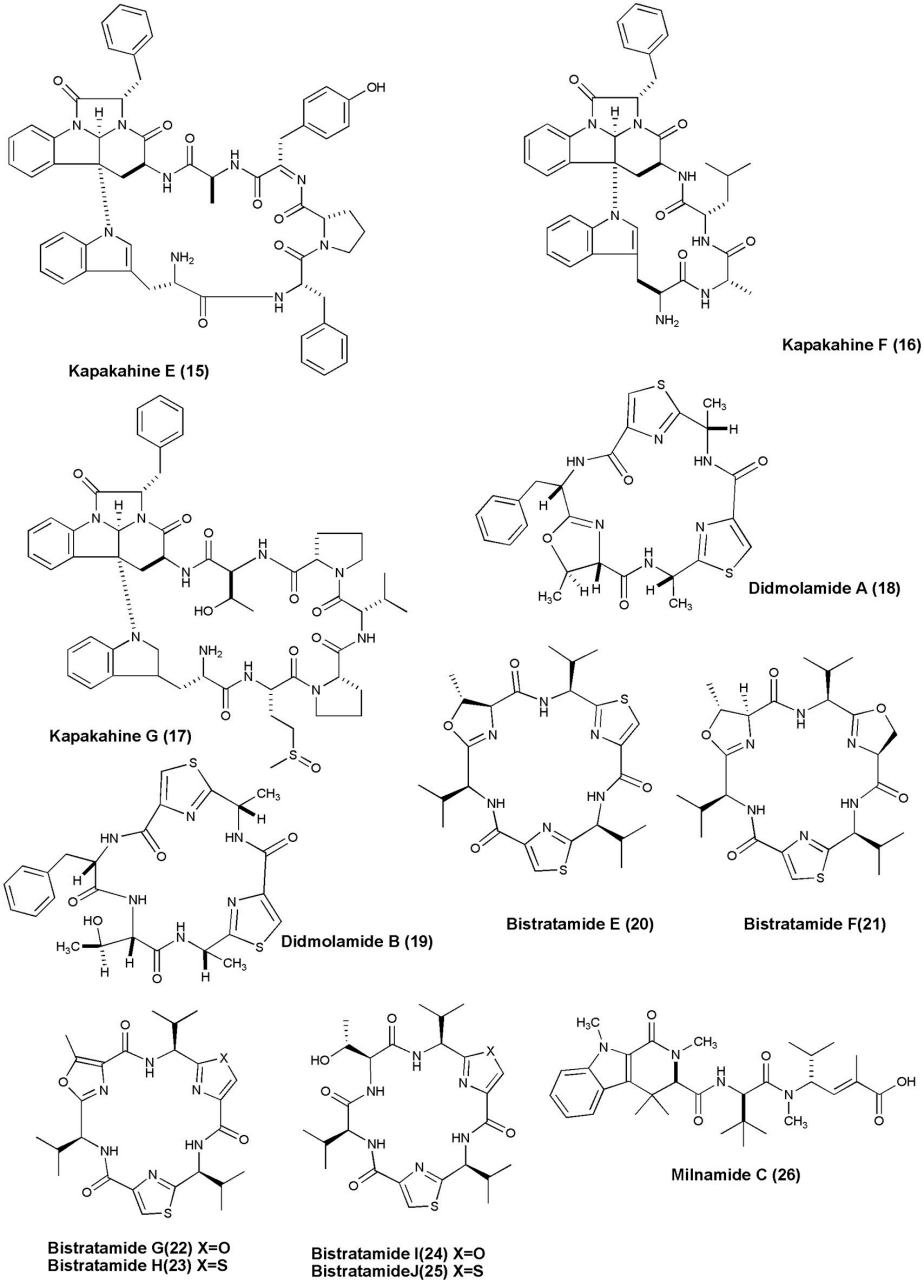
**Structures of anticancer non-ribosomal peptides (15–26)**.

**Figure 3:**
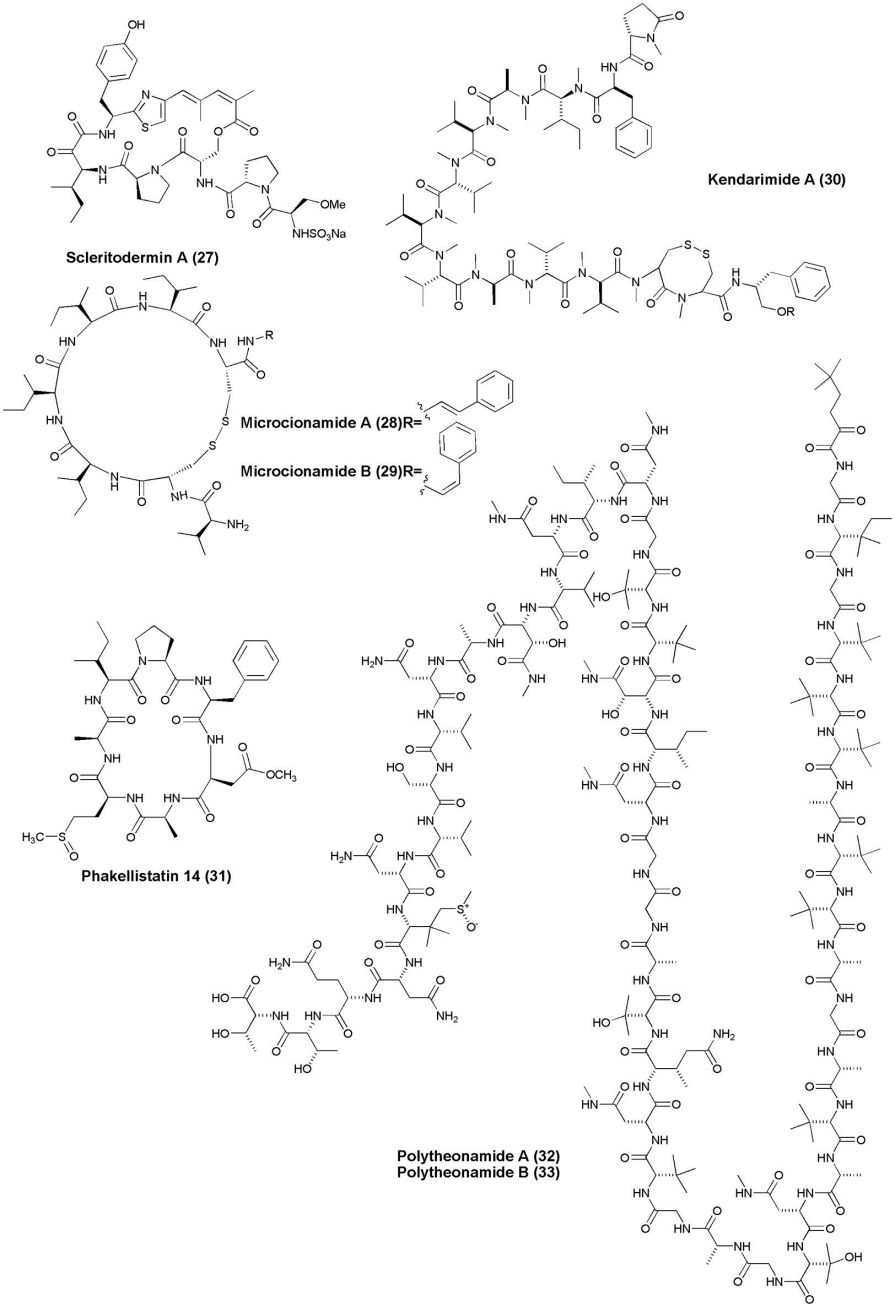
**Structures of anticancer non-ribosomal peptides (27–33)**.

**Figure 4:**
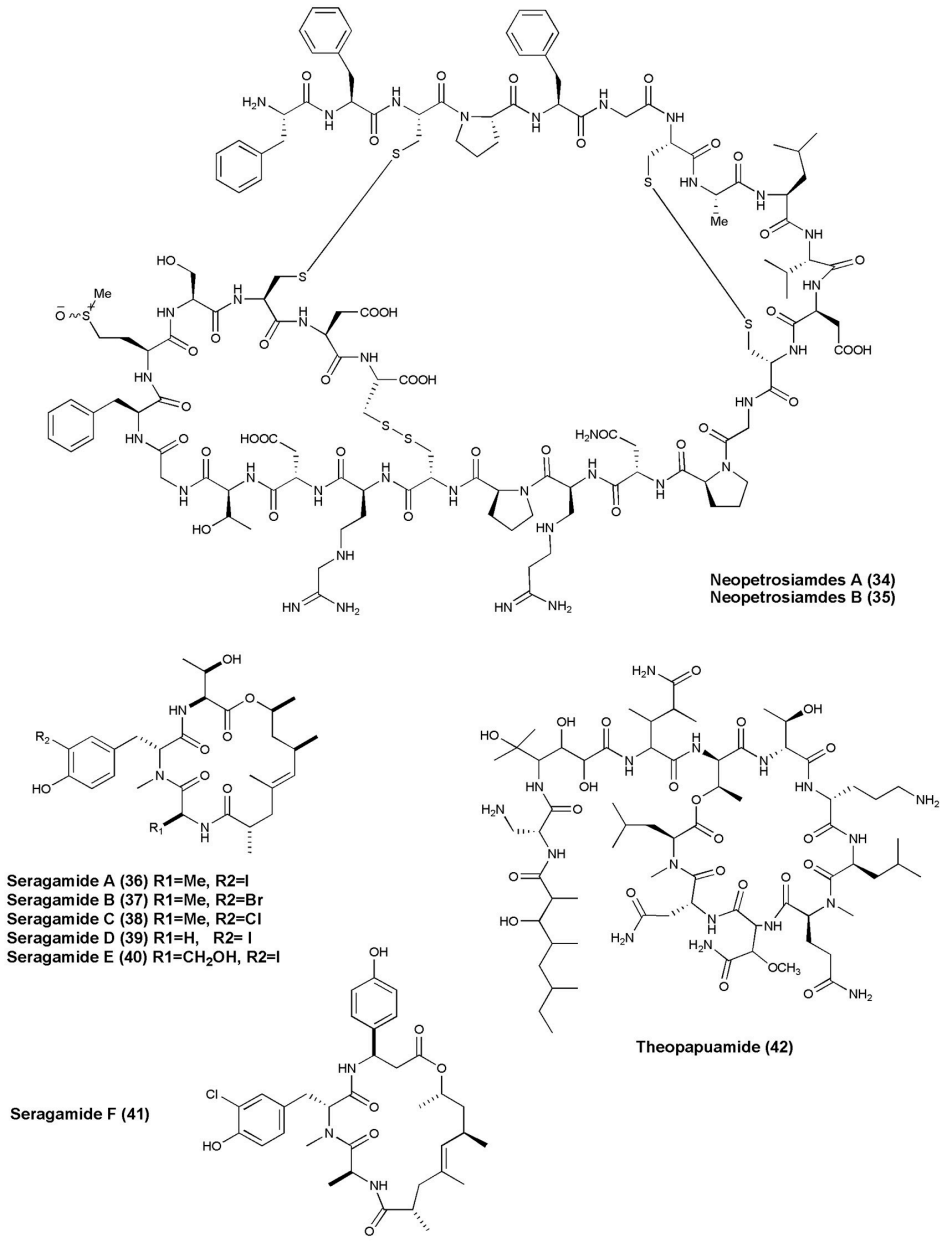
**Structures of anticancer non-ribosomal peptides (34–42)**.

**Figure 5:**
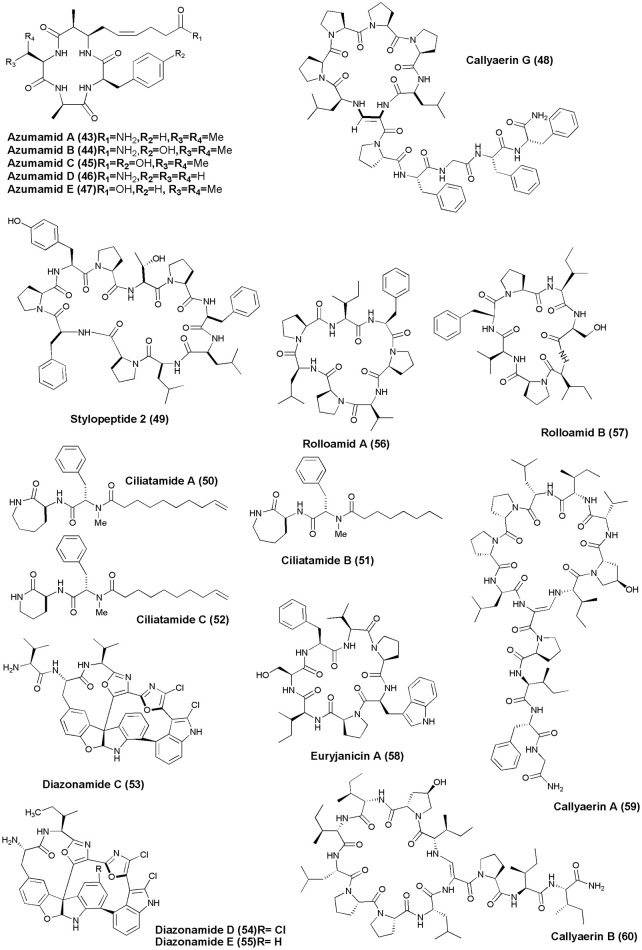
**Structures of anticancer non-ribosomal peptides (43–60)**.

**Figure 6:**
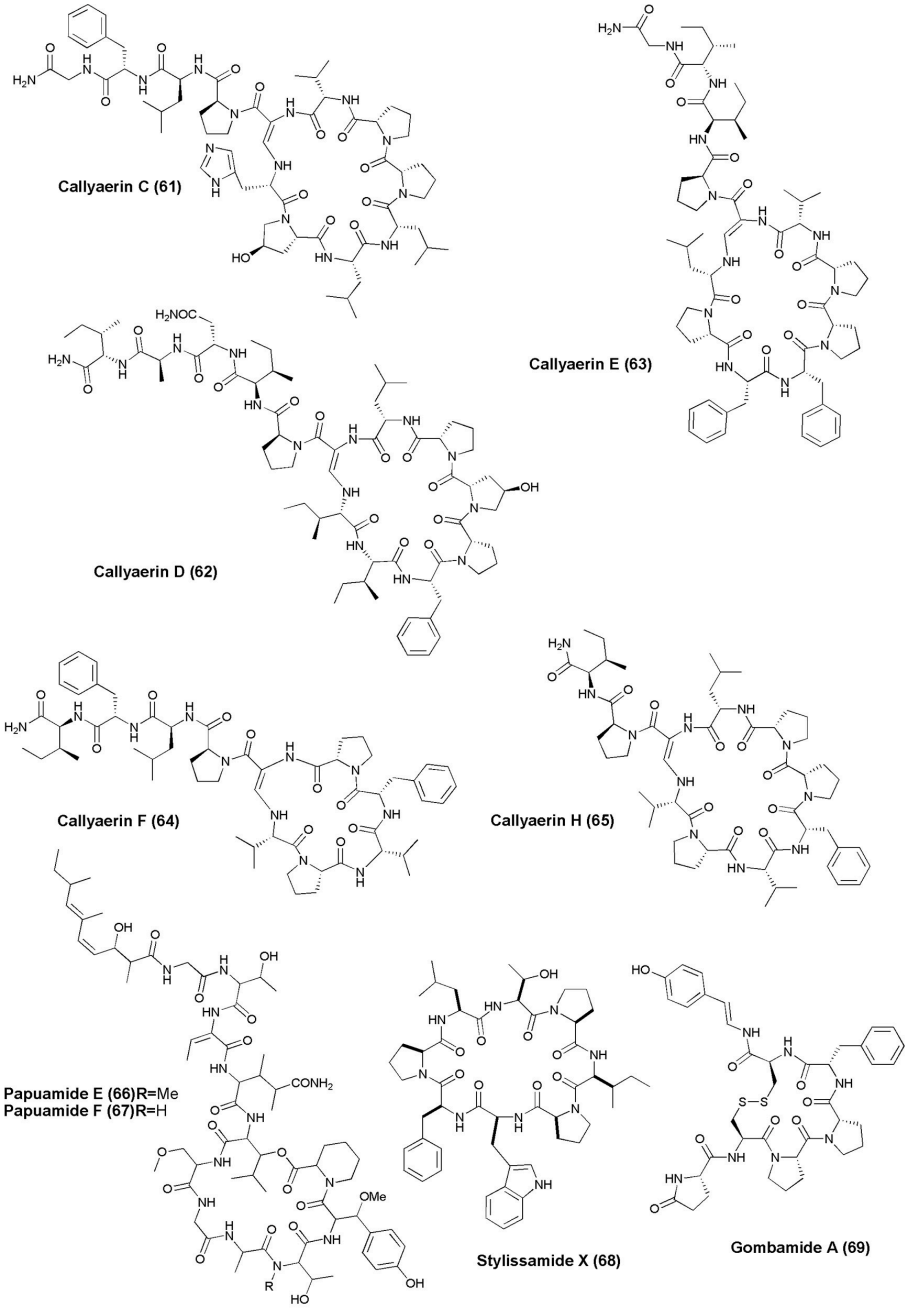
**Structures of anticancer non-ribosomal peptides (61–69)**.

A cyclic peptide, Prepatellamide A **(4)** was isolated from the cytotoxic extracts of *L. patella*. The crude extract of this ascidian showed cytotoxicity against P388 murine leukemia cell lines with IC_50_ = ~5 μg/mL (Fu et al., 2000). Naturally occurring depsipeptides, Tamandarins A and B **(5 and 6)** were discovered from a Brazilian ascidian of the family *Didemnidae* and were cytotoxic against various human cancer cell lines including pancreatic carcinoma BX-PC3, IC_50_ = 1.79, 2.00 μg/mL, prostatic cancer DU-145, IC_50_ = 1.36, 1.53 μg /mL, head and neck carcinoma UMSCC10b, IC_50_ = 0.99, 1.7 6 μg/mL, respectively (Vervoort et al., 2000). *Microscleroderma* sp. from Palau gave 4 new cyclic peptides, Microsclerodermins F–I **(7–10)**, all of which inhibited the growth of *C. albicans* with MIC value of 1.5, 3, 12, and 25 μg per disk, respectively, and also showed cytotoxicity against the HCT-116 cell line with IC_50_ value of 1.8, 2.4, 1.0, and 1.1 μg/mL, respectively (Qureshi et al., 2000).

A histidine-containing proline-rich cyclic heptapeptide, Wainunuamide **(11)**, was isolated from the Fijian marine sponge *S. aurantium*. Compound **(11)** exhibited weak cytotoxic activity against A2780 ovarian tumor and K562 leukemia cancer cells with ID_50_ of 19.15 and 18.36 μg/mL, respectively (Tabudravu et al., 2001). The Australian marine sponge *L. microraphis* was the source of a bioactive cyclic heptapeptide, Leucamide A **(12)**. Compound **(12)** inhibited the growth of the 3 tumor cell lines HM02 (gastric, GI_50_ = 5.2 μg/mL), HepG2 (liver, GI_50_ = 5.9 μg/mL), and Huh7 (liver, GI_50_ = 5.1 μg/mL) (Kehraus et al., 2002). The Fijian collection of marine sponge *S. aurantium* gave a proline-rich cyclic octapeptide, Axinellin C **(13)** (cyclo [Thr1-Val2-Pro3-Trp4-Pro5-Phe6-Pro7-Leu8]). Axinellin C displayed weak cytotoxicity against A2780 ovarian tumor and K562 leukemia cancer cells with ID_50s_ of 13.17 and 4.46 mg/mL, respectively (Tabudravu et al., 2002). The crude extract of a marine sponge *Cymbastela* sp. (Papua New Guinea) gave a cytotoxic peptide Milnamide D **(14)**. Milnamide D was found to exhibit cytotoxicity against HCT-116 at IC_50_ value of 66 nM and inhibition of tubulin polymerization at IC_50_ of 16 μM (Chevallier et al., 2003).

Investigation of marine sponge *C. olemda* yielded 3 new Kapakahines E−G **(15–17)**. Only kapakahines E was found to display moderate cytotoxicity against P388 murine leukemia cells at IC_50_ of 5.0 μg/mL (Nakao et al., 2003). Two novel cyclic hexapeptides, Didmolamides A-B **(18 and 19)** were isolated from ascidian *D. molle* (Madagascar). Both peptides showed mild cytotoxicity against tumor cell lines (Lung—A549, Colon—HT29, and Skin—MEL28) with IC_50_ values of 10–20 μg/mL (Rudi et al., 2003). The Philippines ascidian *L. bistratum* was the source of 6 cyclic hexapeptides, Bistratamides E-J **(20–25)** which showed weak to moderate activity against the human colon tumor (HCT-116) cell line (IC50's: 3, 7.9; 4, 28; 5, 5; 6, 1.7; 7, 9; 8, 1 μg/mL, respectively) (Perez and Faulkner, 2003). Milnamide C **(26)** was isolated from *Auletta* sp., which showed significant activity against MDA-MB-435 breast cancer cells with IC_50_ values of 3.2 × 10^−1^ μg/mL (Sonnenschein et al., 2004).

Scleritodermin A **(27)**, a new cyclic peptide, was isolated from the Lithistid Sponge *S. nodosum*. Scleritodermin A, inhibited tubulin polymerization and demonstrated significant *in vitro* cytotoxicity against a panel of human tumor cell lines (IC50 < 2 μM), including colon (HCT116), ovarian (A2780), and breast carcinoma cell lines SKBR3 (Schmidt et al., 2004). Microcionamides A **(28)** and B **(29)** were isolated from the Philippine marine sponge *Clathria* (Thalysias) *abietina*. Both compounds displayed significant cytotoxicity toward human breast tumor cell lines MCF-7 and SKBR-3 with IC_50_ of 125 and 98 nM for compound **(28)** and 177 and 172 nM for compound **(29)**, respectively (Davis et al., 2004). Methanol extract of an Indonesian marine sponge *Haliclona* sp. gave a linear peptide Kendarimide A **(30)** which reversed P-glycoprotein-mediated multi-drug resistance in mammalian cells (Aoki et al., 2004).

Cycloheptapeptide, Phakellistatin 14 **(31)**, was isolated from *Phakellia* sp., (Chuuk, Federated States of Micronesia). Compound **(31)** exhibited cytotoxicity against the murine lymphocytic leukemia P388 cell line at ED_50_ of 5 μg/mL (Pettit and Tan, 2005). The marine sponge *T. swinhoei* was found to produce highly cytotoxic polypeptides Polytheonamides A and B **(32–33)** with 48 amino acid residues. Both compounds were found to be cytotoxic against P388 murine leukemia cells with IC_50_ values of 78, 68 pg/mL, respectively (Hamada et al., 2005). Two diastereomeric tricyclic peptides Neopetrosiamdes A **(34)** and B **(35)** have been isolated from the marine sponge *Neopetrosia* sp. collected in Papua New Guinea. These peptides inhibited amoeboid invasion of human tumor cells at 6 μg/mL (Williams et al., 2005). Six new depsipeptides, Seragamides A–F **(36–41)** were isolated from sponge *S. japonicas* (Okinawan). Except seragamide F, all seragamides have showed multinuclei formation in NBT-T2 cells at 0.01, 0.02, 0.01, 0.01, and 0.04 mg/mL, respectively. Compound **(36)** also promotes the polymerization of G-actin and stabilizes F-actin filaments (Tanaka et al., 2006). *Theonella swinhoei* from Papua New Guinea gave a cyclic depsipeptide, Theopapuamide **(42)**. This peptide contains several unusual amino acid residues such as β-methoxyasparagine, 4-amino-5-methyl-2, 3, 5-trihydroxy-hexanoic acid, and also contains an amide linked fatty acid moiety, 3-hydroxy-2, 4, 6-trimethyl-octanoic acid (Htoa) with cytotoxicity against CEM-TART (EC_50_ = 0.5 μM) and HCT-116 (EC_50_ = 0.9 μM) cell lines (Ratnayake et al., 2006).

Azumamide A-E **(43–47)** carboxylic acid containing histone deacetylase (HDAC) inhibitor cyclotetrapeptides were recovered from the sponge *M. izuensis*. Only compound **(47)** displayed human histone deacetylase inhibitory activity (Maulucci et al., 2007). An Indonesian sponge *C. aerizusa* gave a new cyclic peptide named Callyaerin G **(48)** with cytotoxicity against mouse lymphoma cell line (L5178Y) and HeLa cells with ED_50_(s) of 0.53 and 5.4 ug/mL, respectively (Ibrahim et al., 2008). The Papua New Guinea marine sponge *Stylotella* sp. was found to produce a new proline-rich cyclodecapeptide, Stylopeptide 2 **(49)** which inhibited the growth of BT-549 and HS 578T 2 breast cancer cell lines by 77 and 56%, respectively (Brennan et al., 2008). Bioactive lipopeptides Ciliatamides A-C **(50–52)** were isolated from the deep-sea sponge *A. ciliate*. Ciliatamides A-B have showed anti-leishmanial activity at 10 μg/mL with 50 and 45.5% growth inhibition, respectively. Ciliatamides A-C also inhibited growth of HeLa cells with IC_50_ values of 50, 4.5, and 50 μg/mL, respectively (Nakao et al., 2008).

The marine ascidian *Diazona* sp. (Indonesia) gave 3 new macrocyclic peptides, Diazonamides C–E **(53–55)**. All the isolated peptides displayed moderate cytotoxicity against a panel of 3 human tumor cell lines (IC_50_'s: A549 = 2.2, 2.9, 8.0 μg/mL; HT29 = 1.8, 2.9, 5.2 μg/mL; MDA-MB-231 = 2.2, 3.1, 9.0 μg/mL) (Fernández et al., 2008). Dominican marine sponge *E. laughlini* gave 2 cyclic heptapeptides, Rolloamides A **(56)** and B **(57)**. Rolloamide A displayed significant growth suppression against several cancer cells (prostate, breast, ovarian, glioma, and renal) with IC_50_'s of 0.4–5.8 μM (Williams et al., 2009). Proline-containing cycloheptapeptide, Euryjanicin A **(58)** was extracted from the marine sponge *P. laughlini* (Vicente et al., 2009). Bioassay guided extraction of the sponge *C. aerizusa* (Ambon, Indonesia) revealed 7 new cytotoxic cyclic peptides Callyaerins A–F **(59–64)** and H **(65)**. All peptides have showed cytotoxicity, however, callyaerins E and H exhibited strong activity against the L5178Y lymphoma cell line with ED_50_ values of 0.39 and 0.48 μM, respectively (Ibrahim et al., 2010).

An undescribed sponge of the genus *Melophlus* sp. (Karumolum, Russell Is., Solomon Is.) yielded 2 depsipeptides, Papuamides E **(66)** and F **(67)**, which were cytotoxic against brine shrimp with LD_50_ values between 92 and 106 μg/mL (Prasad et al., 2011). A proline-rich octapeptide Stylissamide X **(68)** isolated from an Indonesian marine sponge of *Stylissa* sp. inhibited HeLa cells in the concentration range 0.1–10 μM through wound-healing assay (Arai et al., 2012). The marine sponge *C. gombawuiensis* collected from Korean waters gave a disulphide-linked hexapeptide, Gombamide A **(69)**. Gombamide A showed weak cytotoxic activity against the K562 and A549 cell lines with LC_50_ values of 6.9 and 7.1 μM, respectively, as well as moderate inhibitory activity against Na+/K+-ATPase with an LC_50_ value of 17.8 μM (Woo et al., 2013).

## Anti-HIV agents (figures 7, 8)

The marine sponge *S. microspinosa* was the source of a cyclic depsipeptide Microspinosamide **(70)**, inhibited HIV-1 infection with an EC_50_ value of approximately 0.2 μg/mL (Rashid et al., 2001). A Papua New Guinea collection of the marine sponge *N. huxleyi* has been shown to produce a new HIV-inhibitory cyclic depsipeptide, Neamphamide A **(71)**. Neamphamide A displayed potent cytoprotective activity against HIV-1 infection with EC_50_ value ~28 nM (Oku et al., 2004). Four cyclic depsipeptides, Mirabamides A–D **(72–75)**, were isolated from the marine sponge *S. mirabilis*. Mirabamides A, C and D inhibited HIV-1 fusion (Mirabamides A IC_50_ values between 40 and 140 nM: Mirabamides C IC_50_ values between 140 nM and 1.3 μM: and Mirabamides D IC_50_ values between 190 nM and 3.9 μM). Mirabamides A–C also inhibited the growth of *B. subtilis* and *C. albicans* at 1–5 μg/disk (Plaza et al., 2007).

**Figure 7:**
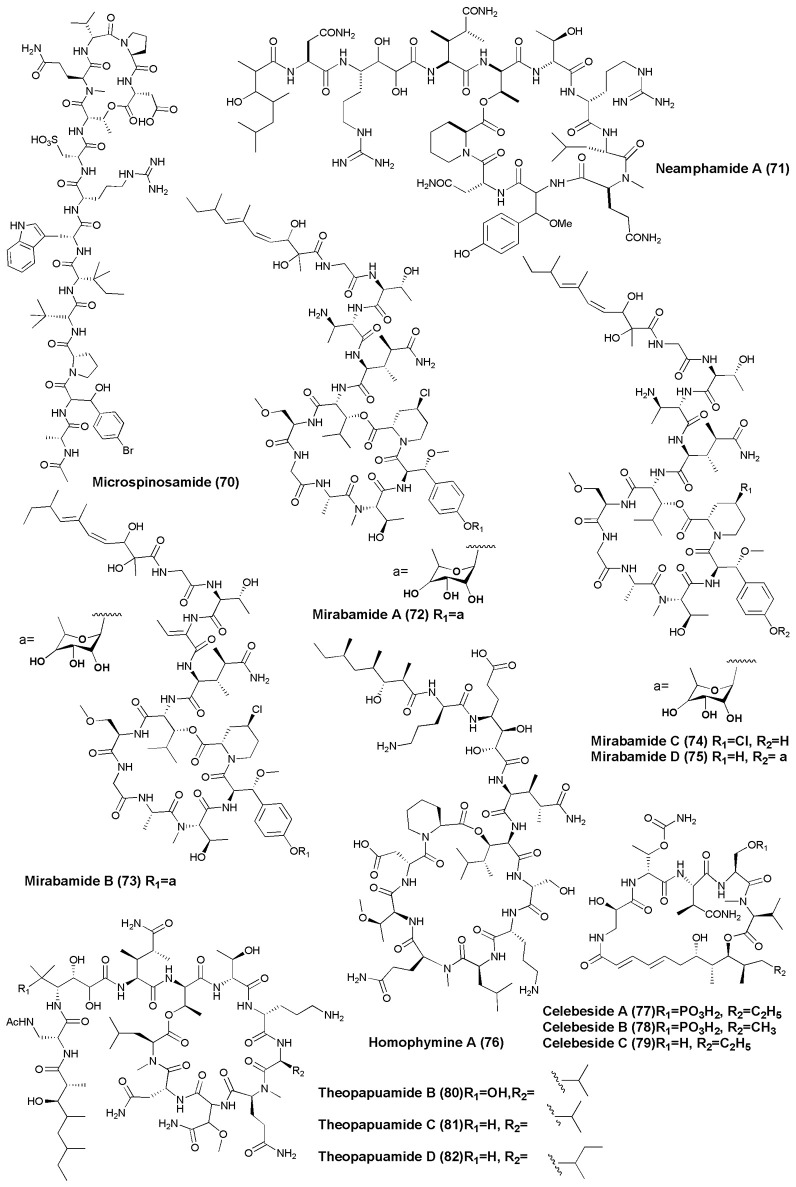
**Structures of non-ribosomal peptides with anti-HIV activity (70–82)**.

**Figure 8:**
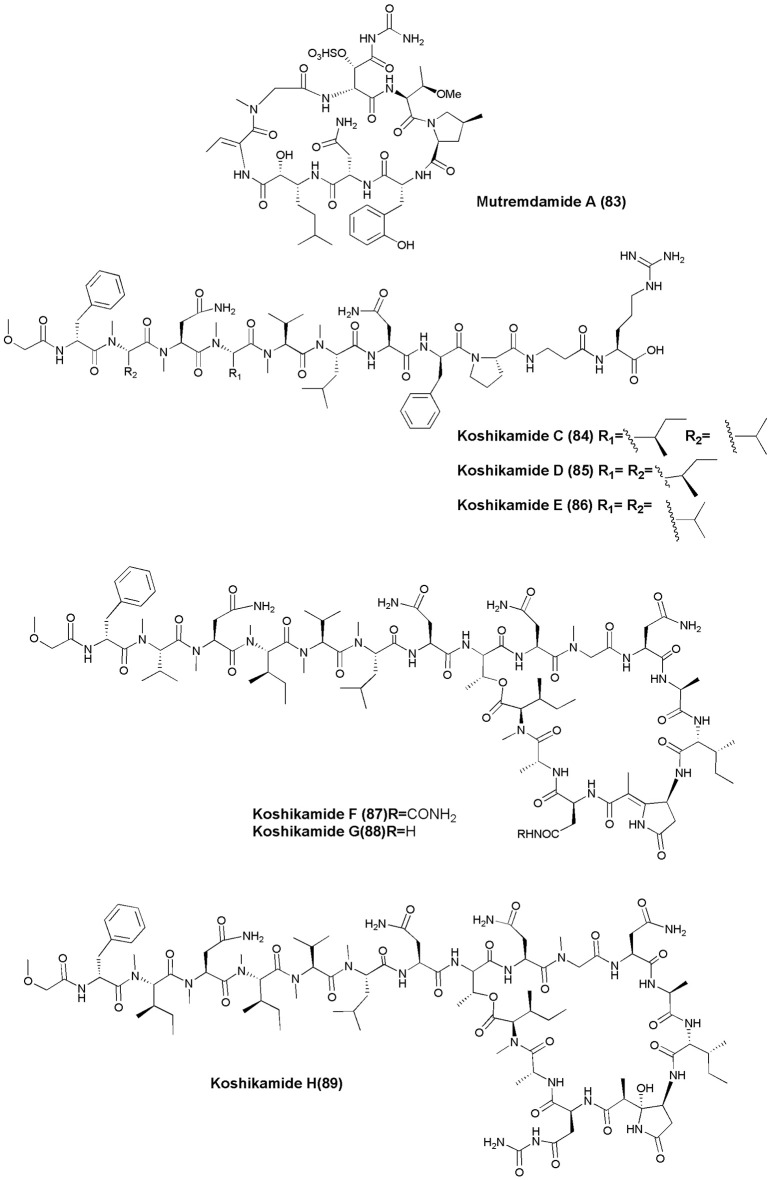
**Structures of non-ribosomal peptides with anti-HIV activity (83–89)**.

The marine sponge *Homophymia* sp. was the source of an anti-HIV cyclodepsipeptide, Homophymine A **(76)**. This peptide inhibited the infection of HIV-1 in PBMC cell line with an IC_50_ of 75 nM (Zampella et al., 2008). Six depsipeptides Celebesides A-C **(77–79)** and Theopapuamides B-D **(80–82)** were isolated from an Indonesian sponge *S. mirabilis*. Compound **(77)** neutralized HIV-1 with an IC_50_ value of 1.9 ± 0.4 μg/mL, while the non-phosphorylated analog Celebeside C was inactive at concentrations as high as 50 μg/mL. Theopapuamides A-C displayed cytotoxicity against human colon carcinoma (HCT-116) cells with IC_50_ values between 2.1 and 4.0 μg/mL, and antifungal activity against wild type and amphotericin B-resistant strains of *C. albicans* at 1–5 μg/disk (Plaza et al., 2008). The deep-water specimens of *T. swinhoei* and *Theonella cupola* (Mutremdiu Reef, Palau) gave sulphated cyclic depsipeptide, Mutremdamide A **(83)** and 6 N-methylated peptides Koshikamides C–H **(84–89)**. Cyclic koshikamides F and H inhibited HIV-1 entry at IC_50_ values of 2.3 and 5.5 μM, respectively, while their linear counterparts were inactive (Plaza et al., 2010).

## Anti-inflammatory NRPs (figure 9)

Marine sponge *S. symbiotica* collected from Biaro Island, Indonesia, alongwith its symbiont marine red alga (Rhodophyta) *Ceratodictyon spongiosum* gave thiazole-containing cyclic heptapeptide, Ceratospongamide **(90)**. Compound **(90)** consists of two l-phenylalanine residues, one (l-isoleucine)-l-methyloxazoline residue, one l-proline residue, and one (l-proline) thiazole residue. The trans-isomer of ceratospongamide exhibits potent inhibition of sPLA2 expression in a cell-based model for anti-inflammation at ED_50_ 32 nM (Tan et al., 2000). Two cyclic depsipeptides, Halipeptins A and B **(91,92)** were obtained from marine sponge *Haliclona* sp. Only halipeptins A displayed *in vivo* potent anti-inflammatory activity (mice at the dose of 300 μg/kg [i.p.]) (Randazzo et al., 2001). A Solomon Lithistid sponge *T. swinhoei* was the source of 2 new cyclopeptides Perthamides C and D with potent anti-inflammatory **(93,94)** (Festa et al., 2009). Cyclic peptides, Solomonamides A and B **(95,96)**, were separated out from the marine sponge *T. swinhoei*; however, only compound **(86)** showed anti-inflammatory activity (Festa et al., 2011). The marine sponge *S. massa* produced a cyclic peptide Stylissatin A **(97)** that inhibited nitric oxide production in LPS-stimulated murine macrophage RAW264.7 cells with an IC_50_ value of 87 μM (Kita et al., 2013).

**Figure 9:**
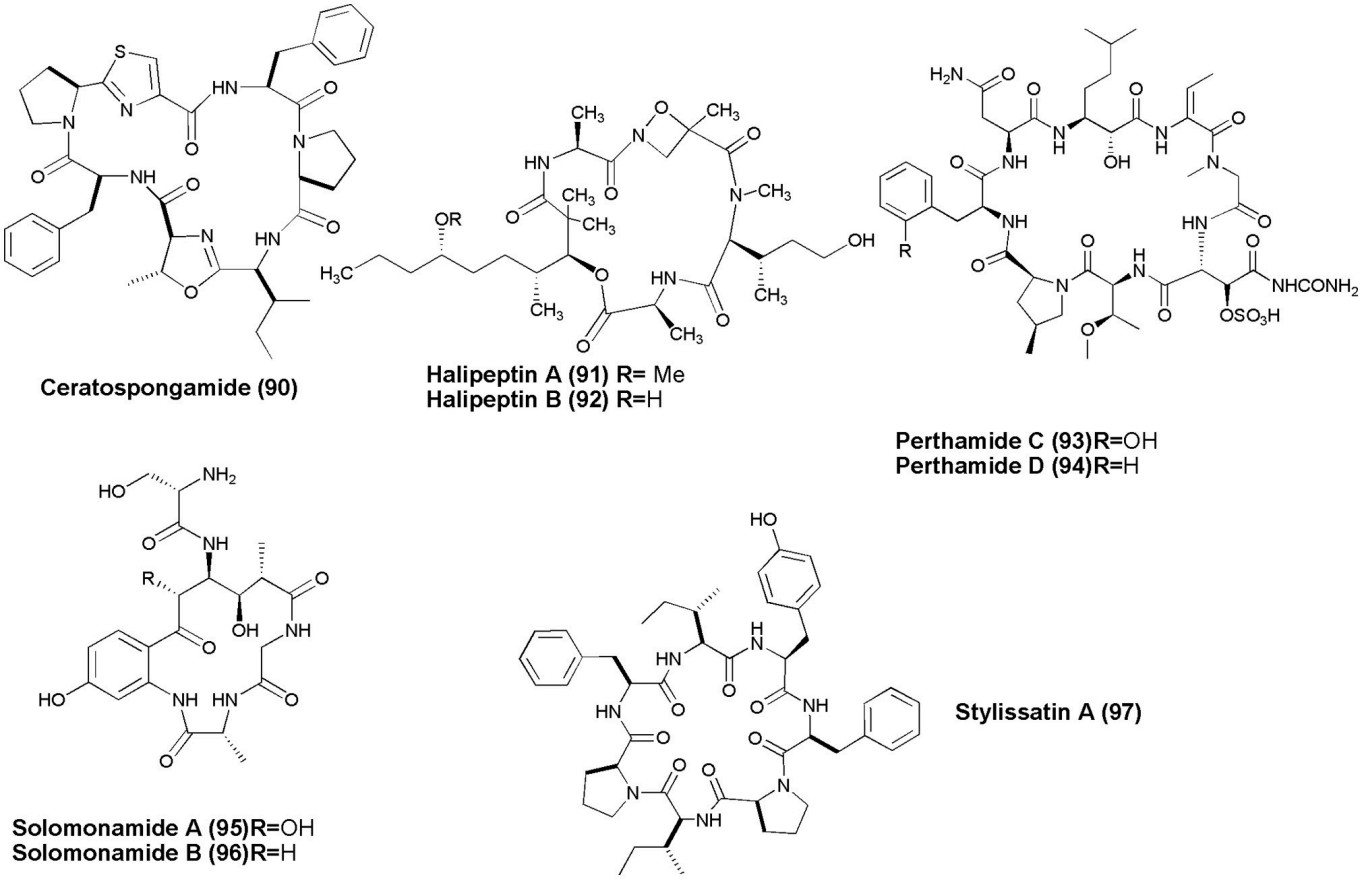
**Structures of non-ribosomal peptides with anti-inflammatory activity (90–97)**.

## Antimicrobial agents (figure 10)

The solitary tunicate, *H. aurantium*, was the source of a novel antimicrobial peptide Dicynthaurin **(98)** (Lee I. H. et al., 2001). An antibacterial depsipeptide, Nagahamide A **(99)**, was discovered from the marine sponge *T. swinhoei* (Okada et al., 2002). An antimicrobial octapeptide Plicatamide **(100)** was isolated from *S. plicata* (Tincu et al., 2003). The marine sponge *Latrunculia* sp., (Vanuatu Islands) was the source of four new antifungal peptides, Callipeltins F–I **(101–104)**. Callipeltins F–I inhibit the growth of *C. albicans* (ATCC24433) with a MIC value of 10^−4^ M (Sepe et al., 2006). Four new peptides, Callipeltins J–M **(105–108)**, were isolated from the marine sponge *Latrunculia* sp. However, only Callipeltins J and K inhibited the growth of *C. albicans* with MIC values of ca. 4^−10^ M (D'Auria et al., 2007). Two new linear tetrapeptides, Citronamides A **(109)** and B **(110)**, were isolated from the Australian sponge *C. astra*. Only citronamides A showed moderate antifungal activity against *Saccharomyces cerevisiae* at MIC value of 8 μg/mL (Carroll et al., 2009).

**Figure 10:**
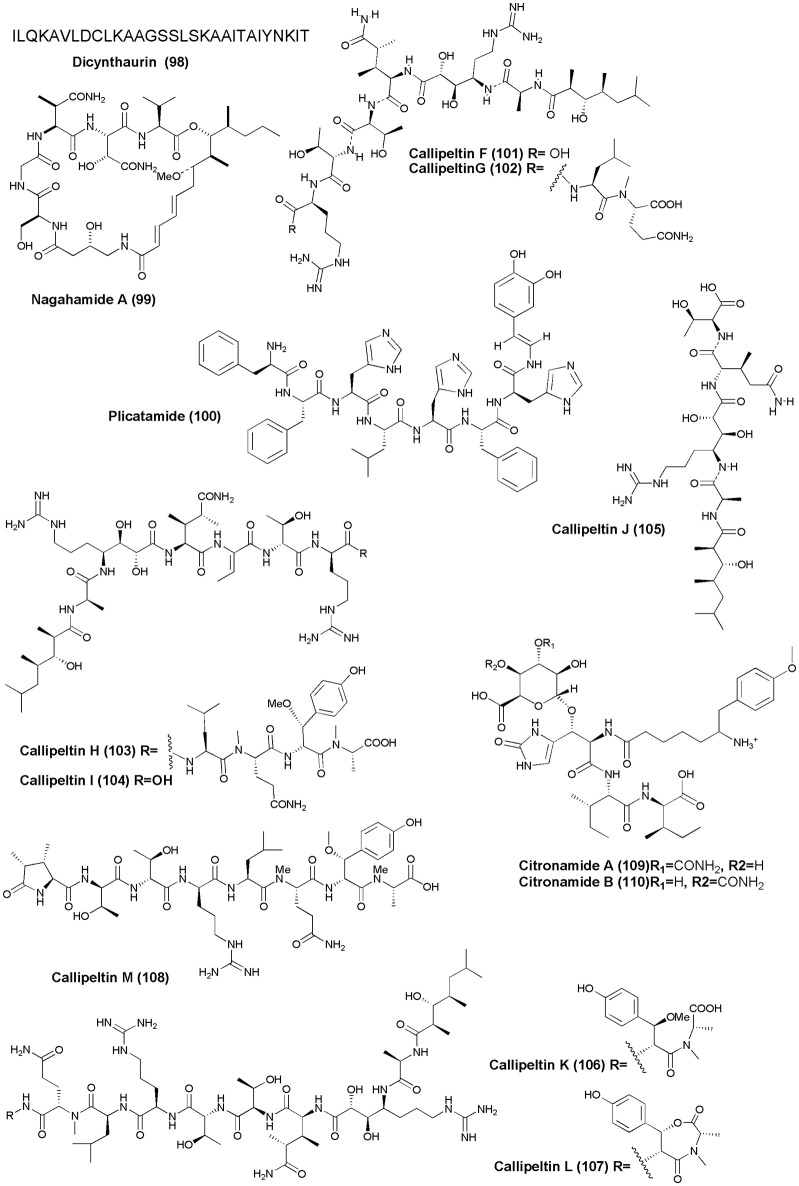
**Structures of antimicrobial non-ribosomal peptides (98–110)**.

## Miscellaneous (figure 11)

A cyclic tripeptide Renieramide **(111)** was isolated from Vanuatu collection of sponge *Reniera* sp. that showed immunomodulating activity in preliminary tests (Ciasullo et al., 2002). Two nematocidal depsipeptides, Phoriospongin A and B **(112 and 113)**, were isolated from Australian marine sponges *Phoriospongia* sp. and *C. bilamellata*. Both compounds have displayed significant nematocidal activity against *H. contortus* (LD_99_ = 100, 194 μg/mL, respectively) (Capon et al., 2002).

**Figure 11:**
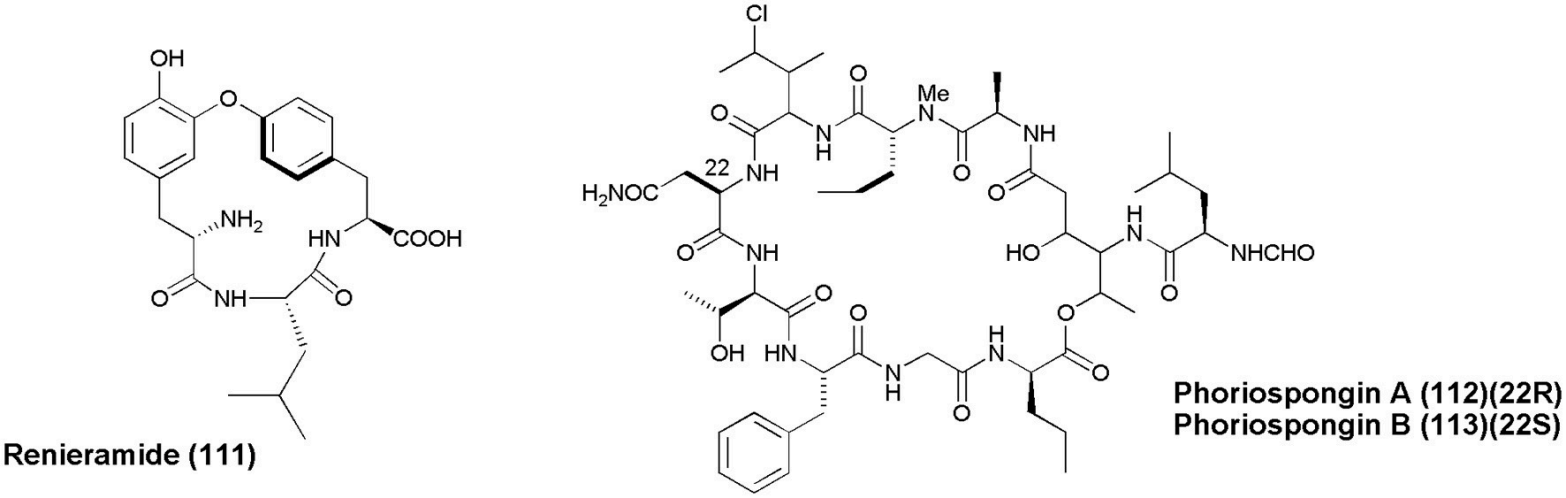
**Structures of non-ribosomal peptides with (111–113)**.

## Biological aspects, challenges, and future perspectives

Like their structural diversity, metabolites produced from marine sponges and tunicates bind to a variety of cellular targets to elicit their effects. Numerous articles published in recent years highlighting the significance of these metabolites in disease control, the details of their biological significance from molecular recognition perspective have been rather scarce. Although some promising leads have been obtained, the discovery of their cellular targets, molecular interactions, and adverse effects are lacking. In cases where the therapeutic potential has been reported, details of a proper screening approach to identify nucleic acid or protein targets are missing. However, some established metabolites from these sources (see Tables 1, 2) and their derivatives have been examined extensively and their molecular targets are varied. One of the earliest examples in this class is FDA-approved drug Ara-C (cytarabine), which is known to elicit anticancer properties by inhibiting the functions of DNA polymerase (Furth and Cohen, 1968), which ultimately results in stalling DNA synthesis. Another FDA approved related compound Ara-A(vidarabine), which is known to have antiviral properties (active against herpes simplex and varicella zoster viruses), targets viral DNA polymerase (Chadwick et al., 1978) by functioning as mimic of natural nucleotides. Both Ara-C and Ara-A resemble natural cytidine and adenine nucleosides where the structural differences are in the sugar components of the two (arabinose vs. deoxyribose). The natural nucleoside mimics Ara-A and Ara-C are easily phosphorylated as their triphosphate derivatives by kinases and act as terminators of DNA synthesis. Ara-A is also known to impede 3′-end processing of pre-mRNAs by inhibiting cleavage and polyadenylation (Ghoshal and Jacob, 1991; Rose and Jacob, 1978).

Several other molecules that are either FDA approved or in early stages of clinical trials have been identified as anticancer agents with microtubules as their primary molecular targets. The predominance of natural metabolites being microtubule binding agents has been hypothesized as evolutionary response to predation by plants and animals (Dumontet and Jordan, 2010). Some of these molecules, such as discodermolide, are among the first non-taxane stabilizers of microtubules (Mooberry et al., 2004). The microtubule stabilizers act by enhancing microtubule polymerization at high concentrations. Discodermolide has been known to bind to tubulin dimers in a stoichiometric ratio. Competitive binding experiments have shown that it blocks taxol binding and is a much stronger binder of microtubules than taxol (Kowalski et al., 1997). The microtubule binding of Tau proteins is interfered by discodermolide (Kar et al., 2003). Similarly, laulimalide showed properties very similar to paclitaxel where it helped in enhancing tubulin assembly (Gapud et al., 2004). However, laulimalide modulation of microtubule assembly in *C. elegans* is dose dependent where it stabilization effects were observed only at concentrations higher than 100 nM (Bajaj and Srayko, 2013).

The antiviral effect of homophymine A has been established by measuring the reverse transcriptase activity in HIV-infected primary peripheral blood mononuclear cells (Zampella et al., 2008). The reverse transcriptase activity is exhibited by 2 classes of molecules: one that directly competes with natural nucleotide triphosphates and the other that either directly blocks the catalytic reactions or by allosteric binding that leads to structural changes in the viral enzyme. Since homophymine A lacks structural features to act as mimics of natural nucleotide triphosphates, it is likely to impede the catalytic activity of the enzyme by direct binding.

A tunicate-derived metabolite trabectedin (ET-743) uses DNA binding to exert its anticancer properties. Trabectin binds to the GC rich regions in the B-DNA where it uses its carbolinamine moiety to form adduct with the exocylic amine (N-2) of guanine (Pommier et al., 1996) and covers 3 base pairs during this process (Marco et al., 2006). Unlike B-DNA minor groove binders, such as Hoechst 33258, which binds snugly along the minor groove curvature with high-affinity (Haq et al., 1997), trabectedin only uses part of its structure to make necessary contacts for the antitumor action (D'Incalci and Galmarini, 2010).

Despite these advances in determining the mode of their binding, a large number of recently discovered metabolites are still not explored to assess it functional capabilities. In the past, well-known anti-retroviral drug zidovudine, which was initially thought to be functionally inert, turned out as excellent therapeutic agent. Such discoveries are possible only when a rational screening design is aimed to asses it full potential as a drug. For example, compounds that have structural regions favorable for protein binding should be screened against all potential protein targets. Similarly, compounds that show preference toward nucleic acid binding should be screened using assays such as competition dialysis that establish a preferential nucleic acid target. Such approaches not only determine the best target for a particular compound but also shed light to its secondary targets, which may be helpful in dealing with toxicity issues. Current target design of marine and tunicate metabolites clearly need to take these approaches.

Some of the metabolites that have weaker binding to a target or have poor bioavailability can be improved by nano-encapsulation techniques. Additionally, DNA binding metabolites can be chemically modified to enhance their affinity using multi-recognition of the target (Willis and Arya, 2010), which has led to remarkable enhancement in the affinity of double,(Arya et al., 2003), triple (Arya and Willis, 2003), and four-stranded DNA helical structures (Ranjan et al., 2013).

## Conclusion

Extreme environment of the ocean plays a vital role in exploring and studying marine bio-resources and their bio-actives. The large biodiversity of the sea serves as a huge resource for developing potential drugs with promising pharmacological activities. The significance of marine-derived secondary metabolites has recently been highlighted by introduction of Prialt and Yondelis to the market. In the past three decades, numerous NRPs with unique chemical structures and varied biological activities have been discovered from marine sponges and tunicates as described in this. Some of these exhibit strong potential to be developed as a new drug. However, none of the NRPs highlighted in this review have been successfully marketed as therapeutics. To translate bioactivity of these important metabolites into therapeutically significant outcomes, it is crucial to further unravel their modes of action and measure their toxicity. Since the majority of these studies have been focused on *in vitro* bioassays and elucidation of the chemical structures only, a complete examination of their biological target selectivity is required. Nevertheless, large-scale production of these NRPs for clinical use is a real challenge. Therefore, environmentally sound and economically feasible alternatives are required. To counter these challenges, many strategies have been established.

Chemical synthesis of NRPs is among the first strategies to be used. However, the structural complexity limits its chemical synthesis and has resulted in only a few successful achievements (e.g., analgesic drug ziconotide; Olivera, 2000). A second strategy uses screening the pharmacological significance of NRPs and subsequently attempting to define the critical pharmacophore that can result in practical drugs based on a marine prototype via chemical synthesis, degradation, modification, or a combination of these. Aquaculture of the source organisms has also been used to secure a sustainable supply of active compounds. However, in most cases, the biomass currently generated is still far from the requirement from an industrial perspective (Mendola, 2000). Identification and large-scale culturing of true producers that are known to thrive within the tissues of marine invertebrates (sponge or tunicate) is an intriguing strategy. However, to date only 5% or less of the symbiotic microbes present in marine specimens can be cultivated under standard conditions. Consequently, molecular approaches such as transfer of biosynthetic gene clusters to a vector suitable for large-scale fermentation could be used to avoid obstacles in culturing symbiotic bacteria. Enzyme technology and solid-phase peptide synthesis offer particularly promising alternatives to generate variety of unique peptides using native peptide as a template. Besides, combinations of chemical synthesis and biosynthetic technologies have potential to accelerate the discovery of novel drugs derived from sponge and their microbial association in future.

## Author contributions

SD reviewed the collected information critically. SA collected the relevant information from various sources including databases like Scifider. AA gave the concept of the work.

### Conflict of interest statement

The authors declare that the research was conducted in the absence of any commercial or financial relationships that could be construed as a potential conflict of interest.
